# Predicting anxiety in cancer survivors presenting to primary care – A machine learning approach accounting for physical comorbidity

**DOI:** 10.1002/cam4.4048

**Published:** 2021-06-02

**Authors:** Markus W. Haun, Laura Simon, Halina Sklenarova, Verena Zimmermann‐Schlegel, Hans‐Christoph Friederich, Mechthild Hartmann

**Affiliations:** ^1^ Department of General Internal Medicine and Psychosomatics Heidelberg University Heidelberg Germany; ^2^ Clinical Psychology and Psychotherapy Ulm University Ulm Baden‐Württemberg Germany; ^3^ Forensic Psychiatric Clinic Ansbach District Hospital Ansbach Germany

**Keywords:** anxiety, cancer survivors, comorbidity, health services research, machine learning, prediction, primary care

## Abstract

**Background:**

The purpose of this study was to explore predictors for anxiety as the most common form of psychological distress in cancer survivors while accounting for physical comorbidity.

**Methods:**

We conducted a secondary data analysis of a large study within the German National Cancer Plan which enrolled primary care cancer survivors diagnosed with colon, prostatic, or breast cancer. We selected candidate predictors based on a systematic MEDLINE search. Using supervised machine learning, we developed a prediction model for anxiety by splitting the data into a 70% training set and a 30% test set and further split the training set into 10‐folds for cross‐validating the hyperparameter tuning step during model selection. We fit six different regression models, selected the model that maximized the root mean square error (RMSE) and fit the selected model to the entire training set. Finally, we evaluated the model performance on the holdout test set.

**Results:**

In total, data from 496 cancer survivors were analyzed. The LASSO model (*α* = 1.0) with weakly penalized model complexity (*λ* = 0.015) slightly outperformed all other models (RMSE = 0.370). Physical symptoms, namely, fatigue/weakness (*β* = 0.18), insomnia (*β* = 0.12), and pain (*β* = 0.04), were the most important predictors, while the degree of physical comorbidity was negligible.

**Conclusions:**

Prediction of clinically significant anxiety in cancer survivors using readily available predictors is feasible. The findings highlight the need for considering cancer survivors’ physical functioning regardless of the degree of comorbidity when assessing their psychological well‐being. The generalizability of the model to other populations should be investigated in future external validations.

## Graphical abstract

Prediction of clinically significant anxiety in cancer survivors using readily available predictors and machine learning is feasible. Clinicians need to consider cancer survivors’ physical functioning regardless of the degree of comorbidity when assessing their psychological well‐being.

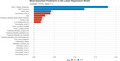

## 1 INTRODUCTION

For most cancer survivors, coping with cancer and its treatment remains a challenge even years after diagnosis.^1^ Coping is often complicated by physical comorbidity. Physical comorbidity is common among cancer survivors, because cancer and comorbidity may share common risk factors, non‐malignant chronic conditions may increase the likelihood of cancer diagnoses, and oncological therapies in turn may contribute to chronic conditions.^2^ A nationwide U.S. survey indicated that 30%–50% of all cancer survivors suffer from physical comorbidity and another survey of breast, prostate, colorectal, and gynecological cancer survivors showed that cancer survivors had on average five comorbid medical diseases.^3^, ^4^

Another major concern in cancer survivors is psychological distress, as over one‐third of all cancer survivors show clinically significant levels of anxiety and/or depression.^5^ Previous research indicates a strong association between psychological distress and physical comorbidity in the general population.^6^ However, it is unclear as to whether physical comorbidity may impact the mental health of cancer survivors. On the one hand, evidence from prospective studies of the general and aging population has shown that adverse physical symptoms and impaired functional status are predictive of psychological distress.^7^, ^8^, ^9^ Additionally, physical comorbidity may increase the financial burden which can also result in psychological distress.^2^ Further, the presence of physical comorbidity may lead to a perception of loss of control, which itself has been found to lead to psychological distress.^10^ On the other hand, it is possible that cancer itself has such a large influence on psychological distress in cancer survivors that physical comorbidity may have virtually no additional impact.^2^ In fact, the severity of physical symptoms, which can vary independent of physical comorbidity, may be a better predictor for psychological distress than the mere number of comorbidities. Evidence for the impact of physical comorbidity on anxiety, the most common type of psychological distress in cancer survivors, is scarce since comorbid conditions are often exclusion criteria in both observational and interventional studies.^11^ In that regard, we conducted a systematic literature search, from inception to May 9, 2021 in MEDLINE, using the search string in Appendix 1. During the screening of 1786 records and relevant cross‐references, we identified 14 studies that examined the relation between physical comorbidity and anxiety in cancer survivors. Nine studies focused on specific tumor entities,^1^, ^12^, ^13^, ^14^, ^15^, ^16^, ^17^, ^18^, ^19^ while five studies sampled patients with heterogeneous tumor entities.^20^, ^21^, ^22^, ^23^, ^24^ These studies generally indicate some form of relation between physical comorbidity and anxiety.^20^, ^21^, ^22^ However, each study usually included only a few predictors often neglecting others such as actual physical symptoms or performance status. Moreover, patients with cancer were often assessed during acute treatment in often tertiary academic centers limiting the generalizability of the findings.

The purpose of this study is to explore potential predictors for anxiety in cancer survivors presenting to primary care accounting for physical comorbidity. Specifically, we apply a machine learning approach using data from a large survey.

## 2 MATERIALS AND METHODS

### 2.1 Source of data

The data were obtained from a large prospective, cross‐sectional observational study within the German National Cancer Plan, entitled “Comparison of two psychosocial cancer care models for rural areas: the P–O–LAND study.”^25^ This study was approved by the Ethics Committee of Heidelberg Medical School (Registration No. S‐300/2013) and is reported in line with the Transparent Reporting of a multivariable prediction model for Individual Prognosis Or Diagnosis (TRIPOD) Statement.^26^

### 2.2 Participants

In the P‐O‐LAND study, we identified all physicians who practiced in the two study regions and provided cancer survivorship care from the mandatory registries of the regional Associations of Statutory Health Insurance Physicians. We initially surveyed these physicians (for results see Zimmermann‐Schlegel et al.^25^). From all responding physicians, we then randomly selected physicians who, in turn, identified and recruited eligible cancer survivors. Cancer survivors were reminded to participate up to four times. We included cancer survivors with a definitive diagnosis of colon, prostatic, or breast cancer and excluded those with cognitive impairment, addiction, psychotic episodes, or suicidality.

### 2.3 Outcome: Clinically significant anxiety

We assessed clinically significant anxiety applying the self‐reported Generalized Anxiety Disorder Scale (GAD)‐2, the brief version of the GAD‐7. Scores range from zero to six. Scores ≥3 indicate clinically significant symptoms of anxiety. At this cut point, the GAD‐2 demonstrated good sensitivity (0.86) and specificity (0.83).^27^ In our sample, the GAD‐2 (Cronbach's *α* = 0.88, 95% CI [0.86–0.90]) showed good reliability.

### 2.4 Predictors

We based the selection of the candidate predictors on the systematic MEDLINE search described above and in more detail in Appendix 2.

### 2.5 Physical comorbidity

We classified the comorbidity status for each cancer survivor applying the Charlson Comorbidity Index (CCI).^28^ The CCI assigns weights of 1, 2, 3, or 6 (i.e., the relative risk of non‐cancer‐related 1‐year mortality rounded to the nearest integer to each of the 13 included comorbid conditions).^29^ Total scores for each patient are derived by summing the weights for each condition. A member of the research team blinded to the goal of this study stratified the comorbid cancer survivors in five comorbidity groups for between‐group comparisons based on their individual CCI sum scores: no comorbidity, very mild comorbidity (comorbidity not enlisted in the CCI, e.g., hypertension), mild comorbidity (CCI sum score of 1), moderate comorbidity (CCI sum score of 2), and severe comorbidity (CCI sum score of 3 to 5). Due to the small number of cancer survivors with sum scores of 3 or higher (13 cancer survivors had a CCI sum score of 3, seven cancer survivors had a sum score of 4, and one participant had a sum score of 5) we collapsed those cancer survivors in one group.

### 2.6 Physical symptoms: Fatigue/weakness, pain, and insomnia

From the psychosomatic complaint subscale of the German version of the Questionnaire on Distress in Cancer Patients—Short Form (QSC‐R10), we assessed the items for physical symptoms fatigue/weakness, pain, and sleep disturbances. In the QSC‐R10, participants rate fatigue/weakness, pain, and insomnia on a scale between 0 and 5. Since we intended to model the distinct relation of each physical symptom (fatigue/weakness, pain, and insomnia) with anxiety, we treated each item as a separate variable instead of calculating the QSC‐R10 sum score.

### 2.7 Additional features

We also included age, disease stage (metastatic disease yes/no), years of education, gender, performance status (WHO‐ECOG), relationship status, time since diagnosis, treatment modality, and tumor location as additional candidate predictors for anxiety in cancer survivors identified in previous work.

### 2.8 Data measurement/sources

For data collection, we asked all eligible cancer survivors to complete an anonymous paper‐and‐pencil self‐reported questionnaire set. A member of the research team blinded to the goal of this study extracted information on physical comorbidity and other medical data (metastatic disease, tumor site, treatment modality, time since diagnosis) from the medical records kept in the primary care practices.

### 2.9 Sample size

In the secondary analysis reported here, we were able to include data of *N* = 496 cancer survivors. Considering the 13 predictors in our model, this sample size allowed us to detect a minimum *R* ^2^ of approximately 0.06 that could be found statistically significant with a power of 0.80.^30^

### 2.10 Statistical analysis

Our primary objective was to predict anxiety (GAD‐2 sum scores) using a combination of all identified predictors in a supervised machine learning approach.^31^ To minimize potential overfitting, we employed different methods, for example, regularizations and penalizations in LASSO regression, out‐of‐bag estimation in Random Forest models, and cross‐validation in all models. We conducted all analyses in R 4.0.3 using the *tidymodels* ecosystem of packages.^32^, ^33^

### 2.11 Developing the reference model

The reference model included all 13 candidate predictors described above.

### 2.12 Feature engineering (data pre‐processing)

First, we computed diagnostics for missing data. Among all predictors, the highest median fraction of missing information (FMI) was 6.5%, among cases the highest FMI was 33.3% (see Table 1). Applying a 50% criterion, all 496 cases were amenable to imputation.^30^, ^34^ Since we did not find sufficient evidence to reject a Missing Completely at Random Process at the 0.05 significance level, we concluded that no systematic missing data process existed for any of the variables. Hence, we used a *k*‐nearest neighbor imputation model to handle missing data for all predictors. Specifically, we selected Gower's distance measure and *k* = 5 contributing neighbors for each predictor.^35^ Second, we converted categorical predictors into binary dummy variables for each level (one‐hot encoding). Third, to correct skewness, we applied Yeo‐Johnson transformation on all numeric variables which all were then centered and scaled. Fourth, we removed any near‐zero variance predictors. Sixth, we split the data randomly into a single training set and a single testing (hold‐out sample) set applying a 70:30 split and the outcome (GAD‐2 score) as a stratum. Finally, we further split the training data into 10‐folds for cross‐validating the hyperparameter tuning step in model selection.

**TABLE 1:** Sample characteristics

	Sample (*N* = 496)	% Missing
Age (Mean [SD])	64.9 [10.95]	0.2
Sex (*n* [%])		
Male	227 [45.8]	0
Education (*n*[%])		
<9 years	285 [54.2]	0.8
≥9 years	227 [45.8]	
Committed relationship (*n* [%])	365 [78.7]	6.5
Weeks since diagnosis (Median [min, max])	112.4 [4.7, 1767.3]	3.0
Tumor localization (*n* [%])		
Colon	204 [41.1]	0
Prostate	102 [20.6]	
Breast	190 [38.3]	
Metastatic disease (*n* [%])	99 [20.3]	1.8
Treatment modality (*n* [%])		
No treatment	226 [47.9]	4.8
Surgery only	41 [8.7]	
Radiation only	10 [2.1]	
Chemotherapy only	46 [9.8]	
Hormone therapy only	97 [20.6]	
Multimodality treatment	52 [11.0]	
ECOG performance status (Mean [SD])	0.3 [0.59]	4.3
Comorbidity status (*n* [%])		
No comorbidity	180 [36.3]	0
Very mild comorbidity^b^	176 [35.5]	
Mild comorbidity^c^	92 [18.6]	
Moderate comorbidity^d^	28 [5.6]	
Severe comorbidity^e^	20 [4.0]	
Physical symptom scores^f^		
Fatigue/weakness (Mean [SD])	1.7 [1.64]	2.2
Pain (Mean [SD])	1.1 [1.42]	1.8
Sleep disturbances (Mean [SD])	1.7 [1.77]	2.2
Psychological distress		
GAD‐2 anxiety score ≥3 (*n* [%])	90 [18.1]	0
PHQ‐2 depression score ≥3 (*n* [%])	82 [16.5]	

Abbreviations: CCI, Charlson comorbidity index; ECOG, Eastern cooperative oncology group; GAD, generalized anxiety disorder scale; PHQ, patient health questionnaire; SD, standard deviation.

^a^ Among those with comorbid conditions in the Charlson Comorbidity Index.

^b^ Participants, with chronic conditions that are not listed in the CCI.

^c^ CCI sum score of 1.

^d^ CCI sum score of 2.

^e^ CCI sum score of 3, 4, and 5.

^f^ Items of the Questionnaire on Distress in Cancer Patients—Short Form.

### 2.13 Model training, hyperparameter tuning, and within‐model comparison

We constructed six machine learning models including an Ordinary Least Square (OLS), Ridge, LASSO, and Elastic Net regression as well as two tree‐based algorithms, namely, Random Forest and XGBoost. To tune the parameters for each of these models, we chose hyperparameter values leading to the best predictive performance metric and performed the *vfold* = 10 cross‐validation with target variable stratification over the hyperparameter grid as a resampling method. Hyperparameter tuning supports the identification of the best value for the bias‐variance trade‐off. In each cross‐validation, randomly selected 70% of the data were used to develop the sub‐models for each model (analysis set) and the remaining 30% to estimate the performance during comparison of sub‐models within a model. Accounting for uniform accuracy across the range of the outcome, we used the root mean squared error (RMSE) as a performance metric (with lower values indicating better accuracy) to determine the optimal hyperparameter configuration and finalize the best sub‐model for each model.

### 2.14 Between‐model approach comparison and computation of predictions

We then trained the best sub‐model for each model fitting them to the entire training dataset to subsequently compare the performance of the best sub‐models of all models. Finally, to attain an independent assessment of the model efficacy for each model, we assessed the out‐of‐sample performance by fitting their best sub‐model to the testing dataset and computing performance of the full model on new (unseen) data.

## 3 RESULTS

### 3.1 Sample characteristics

**FIGURE 1:**
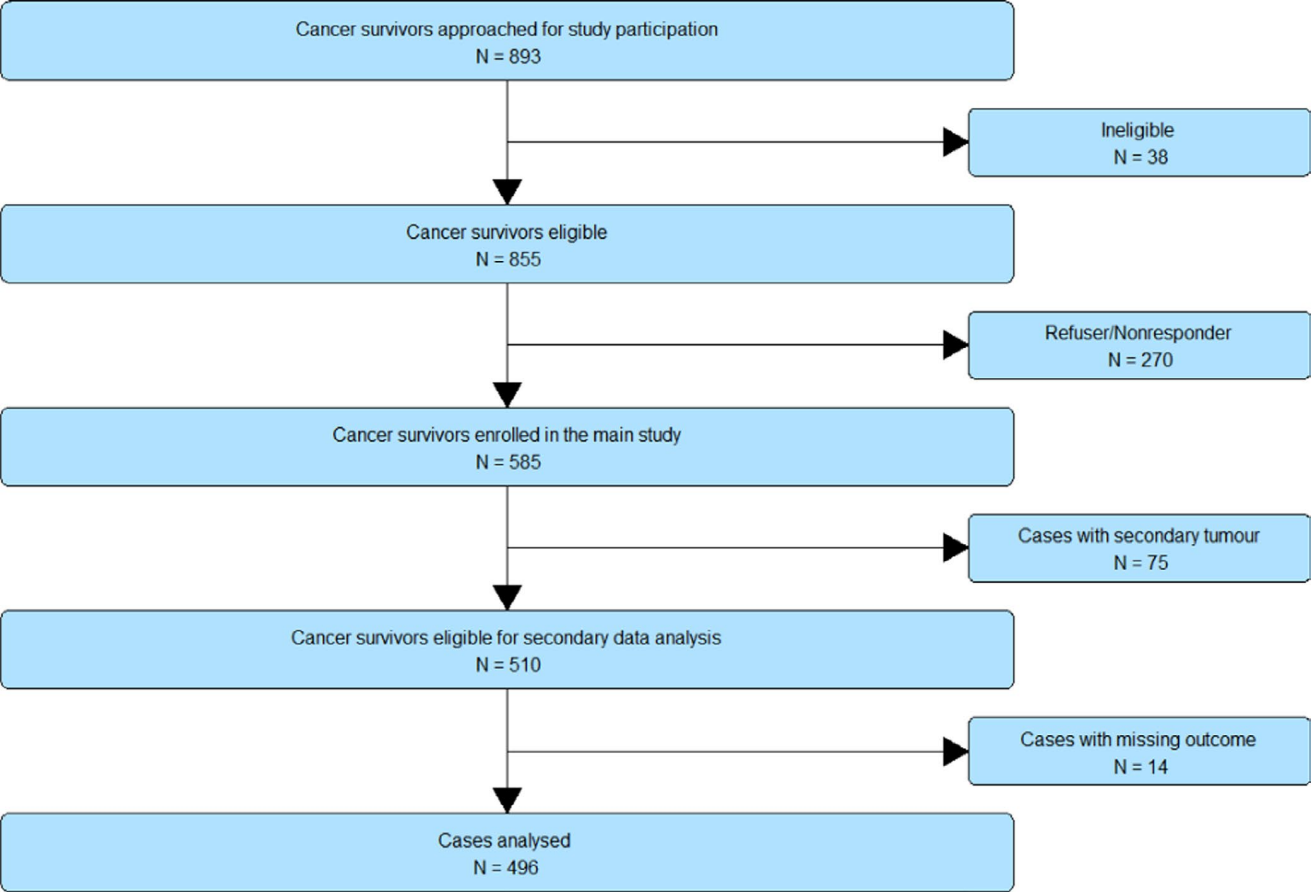
Study flow chart

Please see Figure 1 for the study flowchart. The sample for this study comprised 496 participants. Please see Table 1 for the descriptive characteristics. Notably, 90 participants (17.6%) had a GAD‐2 score ≥ 3 indicating signs of clinically significant anxiety.

### 3.2 Main results

#### 3.2.1 Model selection and performance estimation

**FIGURE 2:**
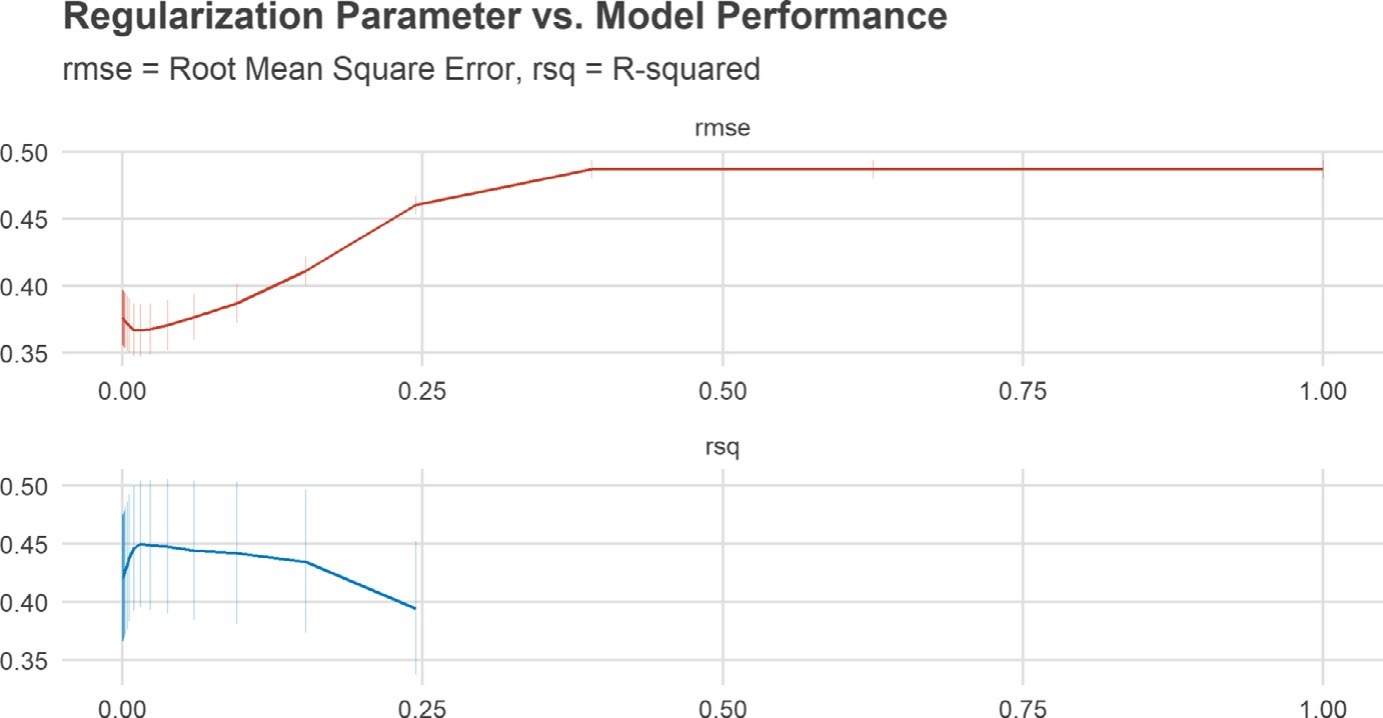
Model performance versus regularization parameter

Cross‐validation showed that the different model approaches for predicting anxiety (OLS, Ridge, LASSO, Elastic Net regression, Random Forest, and XGBoost) varied only slightly with performance metrics ranging from RMSE = 0.370 to RMSE = 0.386 and from *R* ^2^ = 0.370 to *R* ^2^ = 0.427 (Table 2). However, the highly parametric LASSO regression with the regularization parameter *λ* = 0.015 slightly outperformed all other approaches both on the training and the testing dataset (RMSE = 0.370). The LASSO model performance in relation to the regularization parameter *λ* is depicted in Figure 2.

**TABLE 2:** Training and validation performance metrics obtained from 10‐fold cross‐validation for the six best models trained by six machine learning approaches

Model approach	Root mean squared error	*R* ^2^
Ordinary least squares regression	0.372	0.406
Ridge regression	0.373	0.407
LASSO regression	0.370	0.415
Elastic net	0.370	0.413
Random forest	0.0386	0.363
XGBoost	0.373	0.427

### 3.3 Importance of predictors

**FIGURE 3:**
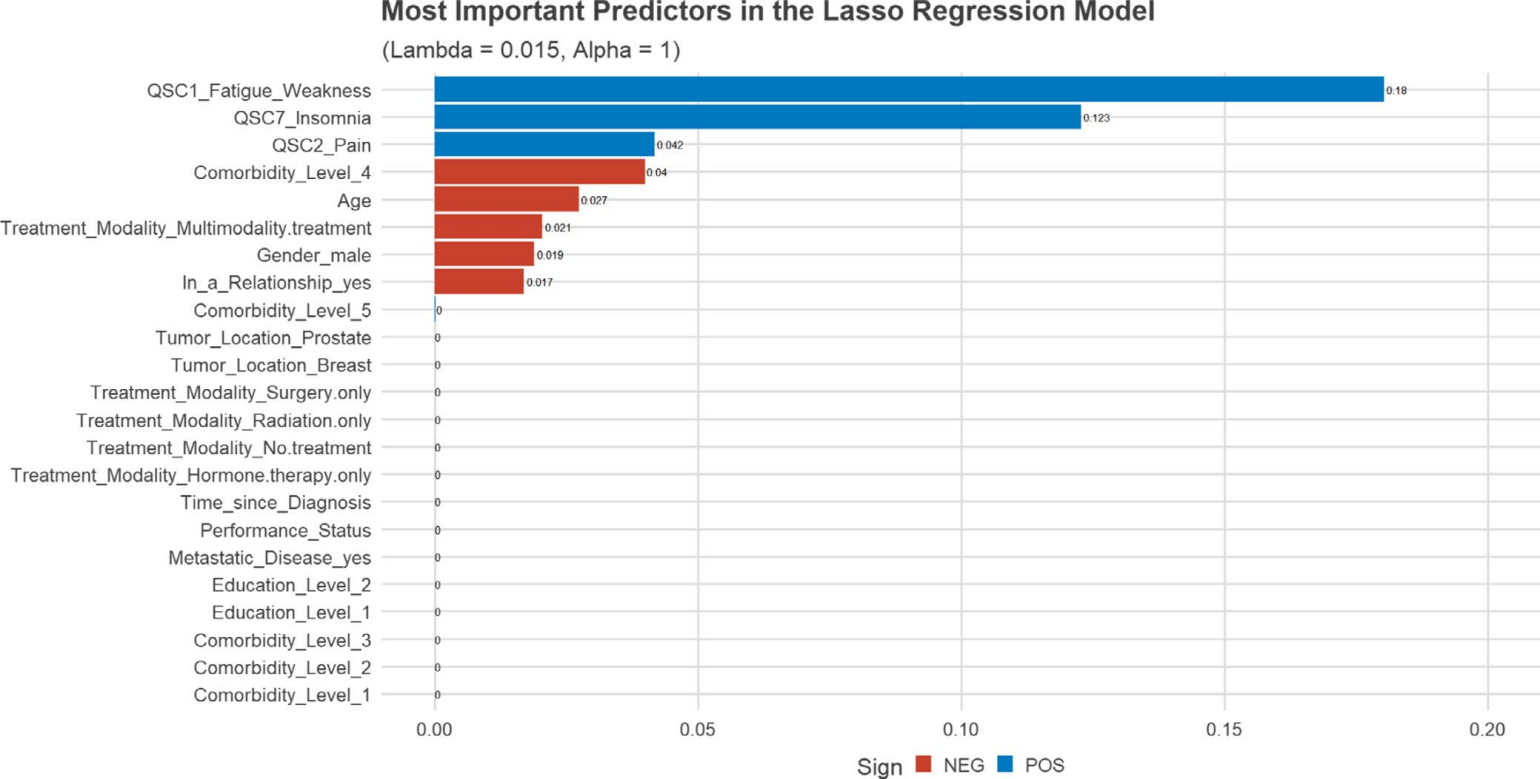
Most important predictors for the LASSO regression model

Importance of all 12 predictors in the LASSO regression model is shown in Figure 3. Fatigue/weakness (*β* = 0.181), insomnia (*β* = 0.122), and pain (*β* = 0.041) emerged as the most important predictors for anxiety. There were no notable correlations between physical symptoms (fatigue/weakness, insomnia, and pain) and the degree of comorbidity (all *r* ≤ 0.08). Notably, age and a moderate degree of comorbidity (CCI group) were predictors for *less* anxiety although of small magnitude.

### 3.4 Model calibration

**FIGURE 4:**
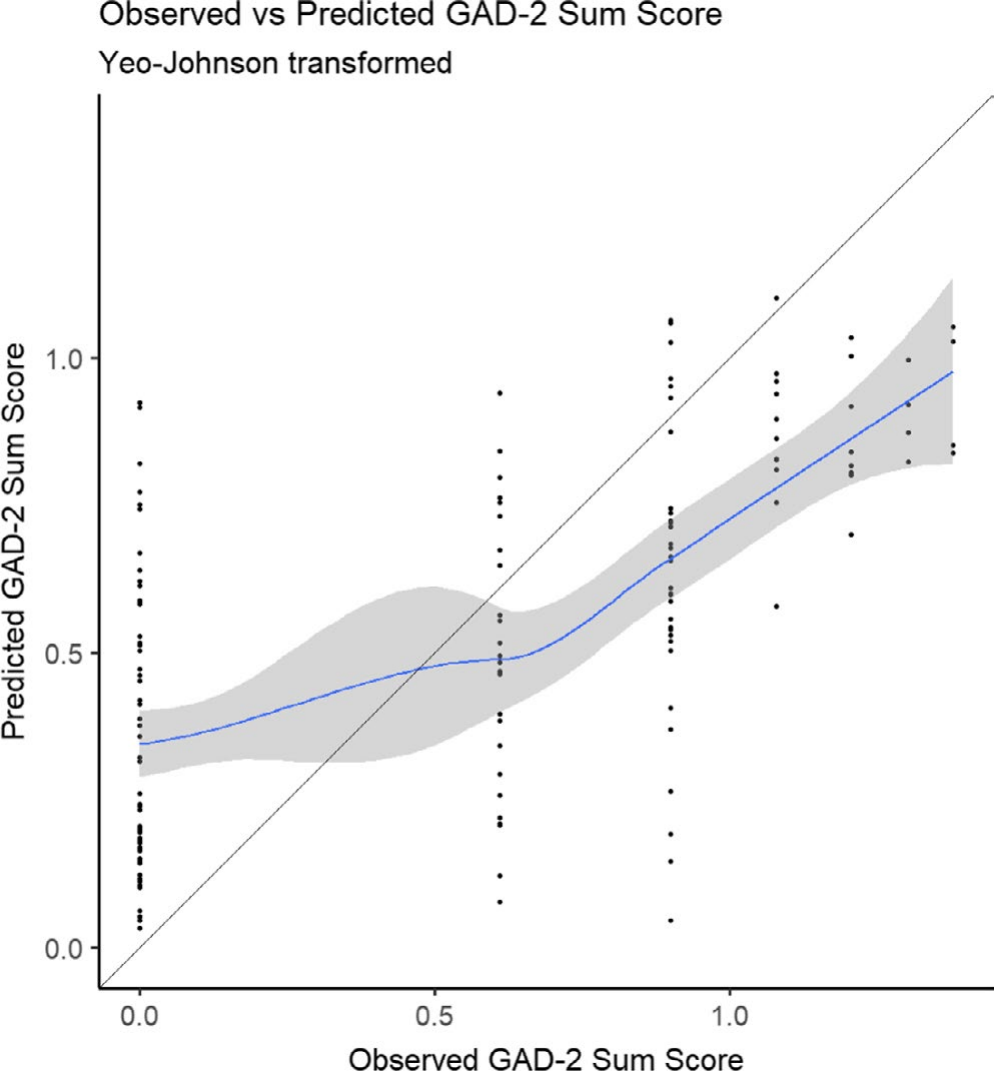
Calibration curve comparing the observed and the predicted Generalized Anxiety Disorder‐2 Scale (GAD‐2) sum scores

The calibration curve in Figure 4 illustrates the agreement between the observed and the predicted scores for anxiety (GAD‐2 sum scores). Notably, higher scores of anxiety were more accurately predicted compared to lower scores.

## 4 DISCUSSION

### 4.1 Key results

This study has been among the first to investigate predictors for anxiety in cancer survivors while controlling for physical symptoms and physical comorbidity. The profound advances in the field of oncology allow many cancer survivors to return to a relatively high level of functioning after having completed active cancer treatment.^36^ However, one in six cancer survivors surveyed in this study experienced clinically significant anxiety which underscores the importance of predictive models to tailor supportive care for this population. The performance of our model indicated that predicting clinically significant anxiety in cancer survivors is challenging, although our model did perform reasonably well for higher scores of anxiety. At any rate, we did show that adverse physical symptoms, such as fatigue/weakness, and insomnia seem to be linked to a higher likelihood of experiencing anxiety. The degree of physical comorbidity had no major role in our predictive model. Rather, our findings indicate that the presence of distressing physical symptoms (such as fatigue/weakness) may contribute to anxiety in cancer survivors to much larger extent compared to type or mere number of comorbid medical diseases. Future work may clarify a potentially protective role of moderate comorbidity, older age, and being in a relationship with respect to anxiety in cancer survivors.

To the best of our knowledge, this is one of the first studies applying a machine learning approach to predict anxiety in cancer survivors after the acute treatment phase and explicitly accounting for comorbidity. The performance of our model was moderate. However, an older study using logistic regression for classification based on the Hospital Anxiety and Depression Scale found a comparable amount of variance explained.^37^ Considering the absence of established theoretical models on the mechanism of anxiety in cancer survivors, our study adds a model based on a comprehensive review of prior work on potential predictors for anxiety. Specifically, we accounted for both sociodemographic and medical characteristics. In contrast to prior work, we did not draw on self‐reports for assessing the medical characteristics, but directly obtained the medical data, including the severity of physical comorbidity, from the health records in the respective primary care practice.^21^, ^22^ We consider this as an important strength, as patients tend to underreport their medical conditions.^38^, ^39^ To evaluate the severity of physical comorbidity, we derived from the common strategy of counting the mere number of chronic medical conditions, but applied the highly valid CCI.^40^ With respect to the most important predictors, we did not find an association between the degree of physical comorbidity and the presence of anxiety, which somewhat contradicts the findings from two previous studies.^20^, ^22^ However, these studies did not model physical symptoms separately, and one may be limited in its cultural generalizability given distinctive aspects of anxiety in Asian populations.^20^, ^41^ In any case, it seems plausible, that people with a larger number of comorbid diseases may have developed profound coping strategies enabling them to better mitigate the impact of a cancer diagnosis and the related treatment.^22^ One prior study reporting associations between unhealthy lifestyles and somatic comorbidity did account for physical symptoms, but focused on elderly cancer survivors and applied the number of chronic medical conditions as a surrogate for the severity of physical comorbidity.^21^ In summary, we provide a cross‐validated, fully tuned model that is optimized with respect to the bias‐variance trade‐off and out‐of‐sample performance. Our findings indicate that the subjective experience of physical symptoms is of greater importance compared to the objective degree of physical comorbidity when evaluating the risk of anxiety in cancer survivors.

This study has some limitations. First, we analyzed cross‐sectional data to identify predictors for anxiety in long‐term cancer survivors accounting for physical comorbidity. Given the interplay of fatigue/weakness, insomnia, and pain, in certain cases anxiety may elicit these symptoms. Nevertheless, our results may facilitate setting prospective cohort studies which are desirable for entangling the complex interplay between physical symptoms, physical comorbidity, and anxiety. In fact, we are aware of only one prospective cohort study addressing this issue. The ACTION Study Group followed patients with cancer from Southeast Asia for up to 12 months after their diagnoses and focused on health‐related quality of life and psychological distress.^20^ The study found that 37% of the participants had at least mild levels of anxiety. This higher rate compared to our sample may be explained by the fact that most of these participants were still in the acute treatment phase. Second, we used the CCI to measure the degree of comorbidity leading to a small number of cancer survivors in the severe comorbidity group. Indeed, given that the CCI stratifies chronic conditions based on a rather serious criterion (the expected 1‐year‐mortality), using this instrument may have affected the generalizability of our findings. Nevertheless, the CCI is still considered to be the comorbidity measure with the highest validity and the distribution of physical comorbidity in our study is comparable to population‐based studies in cancer survivors.^22^, ^40^, ^42^ Third, given that this study included a secondary analysis, we cannot fully rule out left‐out variables error, that is, the omission of predictors that covary with measured predictors but were not excluded in our model as they were not measured in the original study (e.g., functional status).^43^ However, we have tried to minimize the number of left‐out variables so that serious specification error seems unlikely. Fourth, we limited our sampling frame to cancer survivors with three highly prevalent cancer types (i.e., colon, prostatic, and breast cancer). Thus, our findings may be of limited generalizability to cancer survivors diagnosed with other cancer types (e.g., more aggressive ones such as brain, lung, or pancreatic cancer). Fifth, the cross‐cultural generalizability of our findings may also be somewhat limited given the Western perspective we took when analyzing and interpreting the data. For instance, aspects of sexual functioning (including sexual satisfaction) may be a more private matter in Eastern cultures, dealt with more privately, and therefore less reflected in the data. In fact, qualitative studies, preferably using one‐on‐one interviews, may be needed to further elucidate such aspects that potentially differ across cultures. Finally, validation by resampling in our study cannot replace the need for a genuine external validation of the model at another time, other geographical regions, and other health systems. However, resampling did validate the *process* that produced our model.

## 5 CONCLUSIONS

To predict psychological distress in cancer survivors, machine learning‐based approaches allow for the consideration of many predictors and more robust validation of the predictive models. In this study, we found that physical symptoms, namely fatigue/weakness and insomnia, were the predictors of highest practical significance for predicting anxiety. Consequently, clinicians should consistently prioritize the first‐person perspective on physical functioning when evaluating psychological distress in cancer survivors. Indeed, the patients’ subjective experience of their physical and psychological functioning may be the key factor in clarifying patient complexity (overall impact of the different diseases in an individual considering their severity and other health‐related attributes) beyond the mere consideration of comorbidity.^44^

## CONFLICT OF INTEREST

None declared.

## AUTHOR CONTRIBUTIONS

Markus W. Haun: Conceptualization, methodology, formal analysis, investigation, writing–original draft, writing–review and editing, and visualization. Laura Simon: Methodology, formal analysis, writing–original draft, editing, and visualization. Halina Sklenarova: Conceptualization, methodology, and editing. Verena Zimmermann‐Schlegel: Methodology, investigation, and editing. Hans‐Christoph Friederich: Conceptualization, editing, and funding acquisition. Mechthild Hartmann: Conceptualization, methodology, writing–original draft, writing–review and editing, and funding acquisition.

## Data Availability

The data that support the findings of this study are available on request from the corresponding author. The data are not publicly available due to privacy or ethical restrictions.

## APPENDIX 1

### Search String for the Systematic Search in MEDLINE

#### Filters

English, German, Adult: 19+ years, Humans.

#### Search string (including filters)

("neoplasms"[MeSH Terms] AND ("comorbidity"[MeSH Terms] OR "forecast*"[Title/Abstract] OR "predict*"[Title/Abstract] OR "prognos*"[Title] OR "clinical decision rules"[MeSH Terms]) AND ("anxiety"[MeSH Terms] OR "distress"[Title/Abstract])) NOT ("pediatr*"[Title/Abstract] OR "child*"[Title] OR "adolescen*"[Title/Abstract] OR "surger*"[Title] OR "preoperativ*"[Title/Abstract] OR "perioperativ*"[Title/Abstract] OR "transplant*"[Title] OR "caregiv*"[Title] OR "dignit*"[Title/Abstract] OR "colposcop*"[Title/Abstract] OR "smoking cessation"[Title/Abstract] OR "nausea"[Title/Abstract]).

#### Link

https://pubmed.ncbi.nlm.nih.gov/?term=%22neoplasms%22%5BMeSH+Terms%5D+AND+%28%22comorbidity%22%5BMeSH+Terms%5D+OR+%22forecast*%22%5BTitle%2FAbstract%5D+OR+%22predict*%22%5BTitle%2FAbstract%5D+OR+%22prognos*%22%5BTitle%5D+OR+%22clinical+decision+rules%22%5BMeSH+Terms%5D%29+AND+%28%22anxiety%22%5BMeSH+Terms%5D+OR+%22distress%22%5BTitle%2FAbstract%5D%29+NOT+%28pediatr*%5BTitle%2FAbstract%5D+OR+child*%5BTitle%5D+OR+adolescen*%5BTitle%2FAbstract%5D+OR+surger*%5BTitle%5D+OR+preoperativ*%5BTitle%2FAbstract%5D+OR+perioperativ*%5BTitle%2FAbstract%5D+OR+transplant*%5BTitle%5D+OR+caregiv*%5BTitle%5D+OR+dignit*%5BTitle%2FAbstract%5D+OR+colposcop*%5BTitle%2FAbstract%5D+OR+%22smoking+cessation%22%5BTitle%2FAbstract%5D+OR+%22nausea%22%5BTitle%2FAbstract%5D%29&filter=hum_ani.humans&filter=lang.english&filter=lang.german&filter=age.alladult&show_snippets=off&sort=pubdate&size=200

**No. of records:** 1786 (as of May 9th, 2021 and after removal of 1 duplicate record).

## APPENDIX 2

### Predictors for Anxiety in Cancer Survivors (based on MEDLINE Search)

**Table:**

Ability to continue professional work and/or daily life activities
*Negative association with anxiety*
Aass N, Fosså SD, Dahl AA, Aloe TJ. Prevalence of anxiety and depression in cancer patients seen at the Norwegian Radium Hospital. European Journal of Cancer. 1997;33(10):1597–1604. https://doi.org/10.1016/S0959‐8049(97)00054‐3
Wagner T, Augustin M, Blome C, et al. Fear of cancer progression in patients with stage IA malignant melanoma. Eur J Cancer Care. 2018;27(5):e12901. https://doi.org/10.1111/ecc.12901

**Table:**

Age [included in model]
*Negative association with anxiety*
Aass N, Fosså SD, Dahl AA, Aloe TJ. Prevalence of anxiety and depression in cancer patients seen at the Norwegian Radium Hospital. European Journal of Cancer. 1997;33(10):1597–1604. https://doi.org/10.1016/S0959‐8049(97)00054‐3 [<30 or >70 years old]
Acquati C, Kayser K. Predictors of psychological distress among cancer patients receiving care at a safety‐net institution: the role of younger age and psychosocial problems. Support Care Cancer. 2017;25(7):2305–2312. https://doi.org/10.1007/s00520‐017‐3641‐8
Ajaj R, Berlin A, Klaassen Z, et al. Age differences in patient‐reported psychological and physical distress symptoms in bladder cancer patients – a cross sectional study. Urology. 2019;134:154–162. https://doi.org/10.1016/j.urology.2019.08.032
Akechi T, Okamura H, Nishiwaki Y, Uchitomi Y. Psychiatric disorders and associated and predictive factors in patients with unresectable nonsmall cell lung carcinoma: a longitudinal study. Cancer. 2001;92(10):2609–2622. https://doi.org/10.1002/1097‐0142(20011115)92:10<2609::aid‐cncr1614>3.0.co;2‐k
Bisson JI, Chubb HL, Bennett S, Mason M, Jones D, Kynaston H. The prevalence and predictors of psychological distress in patients with early localized prostate cancer: Psychological distress in patients with early localized prostate cancer. BJU International. 2002;90(1):56–61. https://doi.org/10.1046/j.1464‐410X.2002.02806.x
Burgess C, Cornelius V, Love S, Graham J, Richards M, Ramirez A. Depression and anxiety in women with early breast cancer: five year observational cohort study. BMJ. 2005;330(7493):702. https://doi.org/10.1136/bmj.38343.670868.D3
Enns A, Waller A, Groff SL, Bultz BD, Fung T, Carlson LE. Risk factors for continuous distress over a 12‐month period in newly diagnosed cancer outpatients. Journal of Psychosocial Oncology. 2013;31(5):489–506. https://doi.org/10.1080/07347332.2013.822052
Faye‐Schjøll HH, Schou‐Bredal I. Pessimism predicts anxiety and depression in breast cancer survivors: A 5‐year follow‐up study. Psycho‐Oncology. 2019;28(6):1314–1320. https://doi.org/10.1002/pon.5084
Graves KD, Arnold SM, Love CL, Kirsh KL, Moore PG, Passik SD. Distress screening in a multidisciplinary lung cancer clinic: Prevalence and predictors of clinically significant distress. Lung Cancer. 2007;55(2):215–224. https://doi.org/10.1016/j.lungcan.2006.10.001
The ACTION Study Group. Health‐related quality of life and psychological distress among cancer survivors in Southeast Asia: results from a longitudinal study in eight low‐ and middle‐income countries. BMC Med. 2017;15(1):10. https://doi.org/10.1186/s12916‐016‐0768‐2
Harris J, Cornelius V, Ream E, Cheevers K, Armes J. Anxiety after completion of treatment for early‐stage breast cancer: a systematic review to identify candidate predictors and evaluate multivariable model development. Support Care Cancer. 2017;25(7):2321–2333. https://doi.org/10.1007/s00520‐017‐3688‐6
Hinz A, Krauss O, Stolzenburg J‐U, Schwalenberg T, Michalski D, Schwarz R. Anxiety and depression in patients with prostate cancer and other urogenital cancer: A longitudinal study. Urologic Oncology: Seminars and Original Investigations. 2009;27(4):367–372. https://doi.org/10.1016/j.urolonc.2008.02.003
Hipkins J, Whitworth M, Tarrier N, Jayson G. Social support, anxiety and depression after chemotherapy for ovarian cancer: A prospective study. British Journal of Health Psychology. 2004;9(4):569–581. https://doi.org/10.1348/1359107042304542
Hong JS, Tian J. Prevalence of anxiety and depression and their risk factors in Chinese cancer patients. Support Care Cancer. 2014;22(2):453–459. https://doi.org/10.1007/s00520‐013‐1997‐y
Mols F, Schoormans D, de Hingh I, Oerlemans S, Husson O. Symptoms of anxiety and depression among colorectal cancer survivors from the population‐based, longitudinal PROFILES Registry: Prevalence, predictors, and impact on quality of life: Anxiety/Depression in Colorectal Cancer. Cancer. 2018;124(12):2621–2628. https://doi.org/10.1002/cncr.31369
Neilson K, Pollard A, Boonzaier A, et al. A longitudinal study of distress (Depression and anxiety) up to 18 months after radiotherapy for head and neck cancer: Distress 18 months after radiotherapy for head and neck cancer. Psycho‐Oncology. 2013;22(8):1843–1848. https://doi.org/10.1002/pon.3228
Norton TR, Manne SL, Rubin S, et al. Prevalence and predictors of psychological distress among women with ovarian cancer. JCO. 2004;22(5):919–926. https://doi.org/10.1200/JCO.2004.07.028
Osborne RH, Elsworth GR, Hopper JL. Age‐specific norms and determinants of anxiety and depression in 731 women with breast cancer recruited through a population‐based cancer registry. European Journal of Cancer. 2003;39(6):755–762. https://doi.org/10.1016/S0959‐8049(02)00814‐6
Sheppard VB, Harper FWK, Davis K, Hirpa F, Makambi K. The importance of contextual factors and age in association with anxiety and depression in Black breast cancer patients: Anxiety and depression in Black breast cancer patients. Psycho‐Oncology. 2014;23(2):143–150. https://doi.org/10.1002/pon.3382
Thomas BC, Waller A, Malhi RL, et al. A longitudinal analysis of symptom clusters in cancer patients and their sociodemographic predictors. Journal of Pain and Symptom Management. 2014;47(3):566–578. https://doi.org/10.1016/j.jpainsymman.2013.04.007
Weiss Wiesel TR, Nelson CJ, Tew WP, et al. The relationship between age, anxiety, and depression in older adults with cancer: Age, anxiety, and depression in geriatric oncology. Psycho‐Oncology. 2015;24(6):712–717. https://doi.org/10.1002/pon.3638
Yang H, Brand JS, Fang F, et al. Time‐dependent risk of depression, anxiety, and stress‐related disorders in patients with invasive and in situ breast cancer: Depression, anxiety and stress in breast cancer. Int J Cancer. 2017;140(4):841–852. https://doi.org/10.1002/ijc.30514
*Positive association with anxiety*
Abrahamsen AF, Loge JH, Hannisdal E, Holte H, Kvaløy S. Socio‐medical situation for long‐term survivors of Hodgkin's disease: a survey of 459 patients treated at one institution. European Journal of Cancer. 1998;34(12):1865–1870. https://doi.org/10.1016/S0959‐8049(98)00269‐X
Jacob L, Bleicher L, Kostev K, Kalder M. Prevalence of depression, anxiety and their risk factors in German women with breast cancer in general and gynecological practices. J Cancer Res Clin Oncol. 2016;142(2):447–452. https://doi.org/10.1007/s00432‐015‐2048‐5
Kim SH, Kang S, Kim Y‐M, et al. Prevalence and predictors of anxiety and depression among cervical cancer survivors in korea. Int J Gynecol Cancer. 2010;20(6):1017–1024. https://doi.org/10.1111/IGC.0b013e3181e4a704

**Table:**

Baseline anxiety
*Positive association with anxiety*
Boyes AW, Girgis A, D’Este CA, Zucca AC, Lecathelinais C, Carey ML. Prevalence and predictors of the short‐term trajectory of anxiety and depression in the first year after a cancer diagnosis: a population‐based longitudinal study. JCO. 2013;31(21):2724–2729. https://doi.org/10.1200/JCO.2012.44.7540
Couper JW, Love AW, Duchesne GM, et al. Predictors of psychosocial distress 12 months after diagnosis with early and advanced prostate cancer. Medical Journal of Australia. 2010;193(S5). https://doi.org/10.5694/j.1326‐5377.2010.tb03930.x
Faye‐Schjøll HH, Schou‐Bredal I. Pessimism predicts anxiety and depression in breast cancer survivors: A 5‐year follow‐up study. Psycho‐Oncology. 2019;28(6):1314–1320. https://doi.org/10.1002/pon.5084
Gray NM, Hall SJ, Browne S, et al. Predictors of anxiety and depression in people with colorectal cancer. Support Care Cancer. 2014;22(2):307–314. https://doi.org/10.1007/s00520‐013‐1963‐8
Iwatani T, Matsuda A, Kawabata H, Miura D, Matsushima E. Predictive factors for psychological distress related to diagnosis of breast cancer: A longitudinal prospective study of psychological issue and predictors. Psycho‐Oncology. 2013;22(3):523–529. https://doi.org/10.1002/pon.3023
Neilson KA, Pollard AC, Boonzaier AM, et al. Psychological distress (Depression and anxiety) in people with head and neck cancers. Medical Journal of Australia. 2010;193(S5). https://doi.org/10.5694/j.1326‐5377.2010.tb03928.x

**Table:**

Disease stage [included in model]
*Positive association with anxiety*
The ACTION Study Group. Health‐related quality of life and psychological distress among cancer survivors in Southeast Asia: results from a longitudinal study in eight low‐ and middle‐income countries. BMC Med. 2017;15(1):10. https://doi.org/10.1186/s12916‐016‐0768‐2
Iwatani T, Matsuda A, Kawabata H, Miura D, Matsushima E. Predictive factors for psychological distress related to diagnosis of breast cancer: A longitudinal prospective study of psychological issue and predictors. Psycho‐Oncology. 2013;22(3):523–529. https://doi.org/10.1002/pon.3023
Jacob L, Bleicher L, Kostev K, Kalder M. Prevalence of depression, anxiety and their risk factors in German women with breast cancer in general and gynecological practices. J Cancer Res Clin Oncol. 2016;142(2):447–452. https://doi.org/10.1007/s00432‐015‐2048‐5
Kugaya A, Akechi T, Okuyama T, et al. Prevalence, predictive factors, and screening for psychologic distress in patients with newly diagnosed head and neck cancer. Cancer. 2000;88(12):2817–2823. https://doi.org/10.1002/1097‐0142(20000615)88:12<2817::aid‐cncr22>3.0.co;2‐n
Nordin K, Berglund G, Glimelius B, Sjödén P‐O. Predicting anxiety and depression among cancer patients: a clinical model. European Journal of Cancer. 2001;37(3):376–384. https://doi.org/10.1016/S0959‐8049(00)00398‐1
Norton TR, Manne SL, Rubin S, et al. Prevalence and predictors of psychological distress among women with ovarian cancer. JCO. 2004;22(5):919–926. https://doi.org/10.1200/JCO.2004.07.028

**Table:**

Education [included in model]
*Negative association with anxiety*
Fleer J, Sleijfer D, Hoekstra H, Tuinman M, Klip E, Hoekstra‐Weebers J. Objective and subjective predictors of cancer‐related stress symptoms in testicular cancer survivors. Patient Education and Counseling. 2006;64(1–3):142–150. https://doi.org/10.1016/j.pec.2005.12.009
The ACTION Study Group. Health‐related quality of life and psychological distress among cancer survivors in Southeast Asia: results from a longitudinal study in eight low‐ and middle‐income countries. BMC Med. 2017;15(1):10. https://doi.org/10.1186/s12916‐016‐0768‐2
Hopwood P, Sumo G, Mills J, Haviland J, Bliss JM. The course of anxiety and depression over 5 years of follow‐up and risk factors in women with early breast cancer: Results from the UK Standardisation of Radiotherapy Trials (Start). The Breast. 2010;19(2):84–91. https://doi.org/10.1016/j.breast.2009.11.007
Loge J, Abrahamsen A, Ekeberg Ø, Hannisdal E, Kaasa S. Psychological distress after cancer cure: a survey of 459 Hodgkin's disease survivors. Br J Cancer. 1997;76(6):791–796. https://doi.org/10.1038/bjc.1997.464
Mols F, Schoormans D, de Hingh I, Oerlemans S, Husson O. Symptoms of anxiety and depression among colorectal cancer survivors from the population‐based, longitudinal PROFILES Registry: Prevalence, predictors, and impact on quality of life: Anxiety/Depression in Colorectal Cancer. Cancer. 2018;124(12):2621–2628. https://doi.org/10.1002/cncr.31369
Osborne RH, Elsworth GR, Hopper JL. Age‐specific norms and determinants of anxiety and depression in 731 women with breast cancer recruited through a population‐based cancer registry. European Journal of Cancer. 2003;39(6):755–762. https://doi.org/10.1016/S0959‐8049(02)00814‐6

**Table:**

Fatigue [included in model]
*Positive association with anxiety*
Ahlberg K, Ekman T, Wallgren A, Gaston‐Johansson F. Fatigue, psychological distress, coping and quality of life in patients with uterine cancer. J Adv Nurs. 2004;45(2):205–213. https://doi.org/10.1046/j.1365‐2648.2003.02882.x
Brown DJF, McMillan DC, Milroy R. The correlation between fatigue, physical function, the systemic inflammatory response, and psychological distress in patients with advanced lung cancer. Cancer. 2005;103(2):377–382. https://doi.org/10.1002/cncr.20777
Daniëls LA, Oerlemans S, Krol ADG, Creutzberg CL, van de Poll‐Franse LV. Chronic fatigue in Hodgkin lymphoma survivors and associations with anxiety, depression and comorbidity. Br J Cancer. 2014;110(4):868–874. https://doi.org/10.1038/bjc.2013.779
Graves KD, Arnold SM, Love CL, Kirsh KL, Moore PG, Passik SD. Distress screening in a multidisciplinary lung cancer clinic: Prevalence and predictors of clinically significant distress. Lung Cancer. 2007;55(2):215–224. https://doi.org/10.1016/j.lungcan.2006.10.001
Vahdaninia M, Omidvari S, Montazeri A. What do predict anxiety and depression in breast cancer patients? A follow‐up study. Soc Psychiat Epidemiol. 2010;45(3):355–361. https://doi.org/10.1007/s00127‐009‐0068‐7

**Table:**

Gender [included in model]
*Positive association with anxiety*
Aass N, Fosså SD, Dahl AA, Aloe TJ. Prevalence of anxiety and depression in cancer patients seen at the Norwegian Radium Hospital. European Journal of Cancer. 1997;33(10):1597–1604. https://doi.org/10.1016/S0959‐8049(97)00054‐3 (female)
Akechi T, Kugaya A, Okamura H, Nishiwaki Y, Yamawaki S, Uchitomi Y. Predictive factors for psychological distress in ambulatory lung cancer patients. Supportive Care in Cancer. 1998;6(3):281–286. https://doi.org/10.1007/s005200050167 (female)
Enns A, Waller A, Groff SL, Bultz BD, Fung T, Carlson LE. Risk factors for continuous distress over a 12‐month period in newly diagnosed cancer outpatients. Journal of Psychosocial Oncology. 2013;31(5):489–506. https://doi.org/10.1080/07347332.2013.822052 (female)
Hong JS, Tian J. Prevalence of anxiety and depression and their risk factors in Chinese cancer patients. Support Care Cancer. 2014;22(2):453–459. https://doi.org/10.1007/s00520‐013‐1997‐y (female)
Lima MP, Longatto‐Filho A, Osório FL. Predictor variables and screening protocol for depressive and anxiety disorders in cancer outpatients. Kavushansky A, ed. PLoS ONE. 2016;11(3):e0149421. https://doi.org/10.1371/journal.pone.0149421 (female)
Mols F, Schoormans D, de Hingh I, Oerlemans S, Husson O. Symptoms of anxiety and depression among colorectal cancer survivors from the population‐based, longitudinal PROFILES Registry: Prevalence, predictors, and impact on quality of life: Anxiety/Depression in Colorectal Cancer. Cancer. 2018;124(12):2621–2628. https://doi.org/10.1002/cncr.31369 (female)
Neilson KA, Pollard AC, Boonzaier AM, et al. Psychological distress (Depression and anxiety) in people with head and neck cancers. Medical Journal of Australia. 2010;193(S5). https://doi.org/10.5694/j.1326‐5377.2010.tb03928.x (male)
Thomas BC, Waller A, Malhi RL, et al. A longitudinal analysis of symptom clusters in cancer patients and their sociodemographic predictors. Journal of Pain and Symptom Management. 2014;47(3):566–578. https://doi.org/10.1016/j.jpainsymman.2013.04.007 (female)
Wagner T, Augustin M, Blome C, et al. Fear of cancer progression in patients with stage IA malignant melanoma. Eur J Cancer Care. 2018;27(5):e12901. https://doi.org/10.1111/ecc.12901 (female)

**Table:**

Insomnia [included in model]
*Positive association with anxiety*
Bleiker EMA, Pouwer F, van der Ploeg HM, Leer J‐WH, Adèr HJ. Psychological distress two years after diagnosis of breast cancer: frequency and prediction. Patient Education and Counseling. 2000;40(3):209–217. https://doi.org/10.1016/S0738‐3991(99)00085‐3
Delgado‐Guay M, Yennurajalingam S, Parsons H, Palmer JL, Bruera E. Association between self‐reported sleep disturbance and other symptoms in patients with advanced cancer. Journal of Pain and Symptom Management. 2011;41(5):819–827. https://doi.org/10.1016/j.jpainsymman.2010.07.015
Galiano‐Castillo N, Arroyo‐Morales M, Ariza‐Garcia A, Fernández‐Lao C, Fernández‐Fernández AJ, Cantarero‐Villanueva I. Factors that explain the cancer‐related insomnia. Breast J. 2017;23(4):387–394. https://doi.org/10.1111/tbj.12759
Kim SH, Kang S, Kim Y‐M, et al. Prevalence and predictors of anxiety and depression among cervical cancer survivors in korea. Int J Gynecol Cancer. 2010;20(6):1017–1024. https://doi.org/10.1111/IGC.0b013e3181e4a704

**Table:**

Pain [included in model]
*Positive association with anxiety*
Akechi T, Okamura H, Nishiwaki Y, Uchitomi Y. Psychiatric disorders and associated and predictive factors in patients with unresectable nonsmall cell lung carcinoma: a longitudinal study. Cancer. 2001;92(10):2609–2622. https://doi.org/10.1002/1097‐0142(20011115)92:10<2609::aid‐cncr1614>3.0.co;2‐k
Graves KD, Arnold SM, Love CL, Kirsh KL, Moore PG, Passik SD. Distress screening in a multidisciplinary lung cancer clinic: Prevalence and predictors of clinically significant distress. Lung Cancer. 2007;55(2):215–224. https://doi.org/10.1016/j.lungcan.2006.10.001
Vahdaninia M, Omidvari S, Montazeri A. What do predict anxiety and depression in breast cancer patients? A follow‐up study. Soc Psychiat Epidemiol. 2010;45(3):355–361. https://doi.org/10.1007/s00127‐009‐0068‐7

**Table:**

Performance status [included in model]
*Negative association with anxiety*
Akizuki N, Shimizu K, Asai M, et al. Prevalence and predictive factors of depression and anxiety in patients with pancreatic cancer: a longitudinal study. Jpn J Clin Oncol. 2016;46(1):71–77. https://doi.org/10.1093/jjco/hyv169
Gu M, Hao X, Cong L, Sun J. The prevalence, risk factors, and prognostic value of anxiety and depression in refractory or relapsed acute myeloid leukemia patients of North China: Medicine. 2019;98(50):e18196. https://doi.org/10.1097/MD.0000000000018196
Hong JS, Tian J. Prevalence of anxiety and depression and their risk factors in Chinese cancer patients. Support Care Cancer. 2014;22(2):453–459. https://doi.org/10.1007/s00520‐013‐1997‐y

**Table:**

Previous mental health condition
*Positive association with anxiety*
Harris J, Cornelius V, Ream E, Cheevers K, Armes J. Anxiety after completion of treatment for early‐stage breast cancer: a systematic review to identify candidate predictors and evaluate multivariable model development. Support Care Cancer. 2017;25(7):2321–2333. https://doi.org/10.1007/s00520‐017‐3688‐6
Jacob L, Bleicher L, Kostev K, Kalder M. Prevalence of depression, anxiety and their risk factors in German women with breast cancer in general and gynecological practices. J Cancer Res Clin Oncol. 2016;142(2):447–452. https://doi.org/10.1007/s00432‐015‐2048‐5
Lima MP, Longatto‐Filho A, Osório FL. Predictor variables and screening protocol for depressive and anxiety disorders in cancer outpatients. Kavushansky A, ed. PLoS ONE. 2016;11(3):e0149421. https://doi.org/10.1371/journal.pone.0149421
Loge J, Abrahamsen A, Ekeberg Ø, Hannisdal E, Kaasa S. Psychological distress after cancer cure: a survey of 459 Hodgkin's disease survivors. Br J Cancer. 1997;76(6):791–796. https://doi.org/10.1038/bjc.1997.464

**Table:**

Relationship status [included in model]
*Negative association with anxiety*
Aass N, Fosså SD, Dahl AA, Aloe TJ. Prevalence of anxiety and depression in cancer patients seen at the Norwegian Radium Hospital. European Journal of Cancer. 1997;33(10):1597–1604. https://doi.org/10.1016/S0959‐8049(97)00054‐3
Akechi T, Kugaya A, Okamura H, Nishiwaki Y, Yamawaki S, Uchitomi Y. Predictive factors for psychological distress in ambulatory lung cancer patients. Supportive Care in Cancer. 1998;6(3):281–286. https://doi.org/10.1007/s005200050167
Balderson N, Towell T. The prevalence and predictors of psychological distress in men with prostate cancer who are seeking support. British Journal of Health Psychology. 2003;8(2):125–134. https://doi.org/10.1348/135910703321649114
Burgess C, Cornelius V, Love S, Graham J, Richards M, Ramirez A. Depression and anxiety in women with early breast cancer: five year observational cohort study. BMJ. 2005;330(7493):702. https://doi.org/10.1136/bmj.38343.670868.D3
Carlson LE, Waller A, Groff SL, Giese‐Davis J, Bultz BD. What goes up does not always come down: patterns of distress, physical and psychosocial morbidity in people with cancer over a one year period: Patterns of distress over one year in cancer outpatients. Psycho‐Oncology. 2013;22(1):168–176. https://doi.org/10.1002/pon.2068
Kugaya A, Akechi T, Okuyama T, et al. Prevalence, predictive factors, and screening for psychologic distress in patients with newly diagnosed head and neck cancer. Cancer. 2000;88(12):2817–2823. https://doi.org/10.1002/1097‐0142(20000615)88:12<2817::aid‐cncr22>3.0.co;2‐n
Lima MP, Longatto‐Filho A, Osório FL. Predictor variables and screening protocol for depressive and anxiety disorders in cancer outpatients. Kavushansky A, ed. PLoS ONE. 2016;11(3):e0149421. https://doi.org/10.1371/journal.pone.0149421
Mols F, Schoormans D, de Hingh I, Oerlemans S, Husson O. Symptoms of anxiety and depression among colorectal cancer survivors from the population‐based, longitudinal PROFILES Registry: Prevalence, predictors, and impact on quality of life: Anxiety/Depression in Colorectal Cancer. Cancer. 2018;124(12):2621–2628. https://doi.org/10.1002/cncr.31369

**Table:**

Somatic comorbidity [included in model]
*Positive association with anxiety*
Abuelgasim K, Ahmed G, Alqahtani J, Alayed A, Alaskar A, Malik M. Depression and anxiety in patients with hematological malignancies, prevalence, and associated factors. SMJ. 2016;37(8):877–881. https://doi.org/10.15537/smj.2016.8.14597
Boyes AW, Girgis A, D’Este CA, Zucca AC, Lecathelinais C, Carey ML. Prevalence and predictors of the short‐term trajectory of anxiety and depression in the first year after a cancer diagnosis: a population‐based longitudinal study. JCO. 2013;31(21):2724–2729. https://doi.org/10.1200/JCO.2012.44.7540
The ACTION Study Group. Health‐related quality of life and psychological distress among cancer survivors in Southeast Asia: results from a longitudinal study in eight low‐ and middle‐income countries. BMC Med. 2017;15(1):10. https://doi.org/10.1186/s12916‐016‐0768‐2
Mols F, Schoormans D, de Hingh I, Oerlemans S, Husson O. Symptoms of anxiety and depression among colorectal cancer survivors from the population‐based, longitudinal PROFILES Registry: Prevalence, predictors, and impact on quality of life: Anxiety/Depression in Colorectal Cancer. Cancer. 2018;124(12):2621–2628. https://doi.org/10.1002/cncr.31369
Robbertz AS, Weiss DM, Awan FT, Byrd JC, Rogers KA, Woyach JA. Identifying risk factors for depression and anxiety symptoms in patients with chronic lymphocytic leukemia. Support Care Cancer. 2020;28(4):1799–1807. https://doi.org/10.1007/s00520‐019‐04991‐y
Weiss Wiesel TR, Nelson CJ, Tew WP, et al. The relationship between age, anxiety, and depression in older adults with cancer: Age, anxiety, and depression in geriatric oncology. Psycho‐Oncology. 2015;24(6):712–717. https://doi.org/10.1002/pon.3638

**Table:**

Time since diagnosis [included in model]
*Negative association with anxiety*
Boyes AW, Girgis A, D’Este CA, Zucca AC, Lecathelinais C, Carey ML. Prevalence and predictors of the short‐term trajectory of anxiety and depression in the first year after a cancer diagnosis: a population‐based longitudinal study. JCO. 2013;31(21):2724–2729. https://doi.org/10.1200/JCO.2012.44.7540

**Table:**

Trait anxiety
*Positive association with anxiety*
Bleiker EMA, Pouwer F, van der Ploeg HM, Leer J‐WH, Adèr HJ. Psychological distress two years after diagnosis of breast cancer: frequency and prediction. Patient Education and Counseling. 2000;40(3):209–217. https://doi.org/10.1016/S0738‐3991(99)00085‐3

**Table:**

Treatment modality [included in model]
*Positive association with anxiety*
Chipperfield K, Fletcher J, Millar J, et al. Predictors of depression, anxiety and quality of life in patients with prostate cancer receiving androgen deprivation therapy: Prostate cancer and androgen deprivation therapy. Psycho‐Oncology. 2013;22(10):2169–2176. https://doi.org/10.1002/pon.3269
Enns A, Waller A, Groff SL, Bultz BD, Fung T, Carlson LE. Risk factors for continuous distress over a 12‐month period in newly diagnosed cancer outpatients. Journal of Psychosocial Oncology. 2013;31(5):489–506. https://doi.org/10.1080/07347332.2013.822052
The ACTION Study Group. Health‐related quality of life and psychological distress among cancer survivors in Southeast Asia: results from a longitudinal study in eight low‐ and middle‐income countries. BMC Med. 2017;15(1):10. https://doi.org/10.1186/s12916‐016‐0768‐2
Gu M, Hao X, Cong L, Sun J. The prevalence, risk factors, and prognostic value of anxiety and depression in refractory or relapsed acute myeloid leukemia patients of North China: Medicine. 2019;98(50):e18196. https://doi.org/10.1097/MD.0000000000018196
Hinz A, Krauss O, Stolzenburg J‐U, Schwalenberg T, Michalski D, Schwarz R. Anxiety and depression in patients with prostate cancer and other urogenital cancer: A longitudinal study. Urologic Oncology: Seminars and Original Investigations. 2009;27(4):367–372. https://doi.org/10.1016/j.urolonc.2008.02.003
Hopwood P, Sumo G, Mills J, Haviland J, Bliss JM. The course of anxiety and depression over 5 years of follow‐up and risk factors in women with early breast cancer: Results from the UK Standardisation of Radiotherapy Trials (Start). The Breast. 2010;19(2):84–91. https://doi.org/10.1016/j.breast.2009.11.007
Loge J, Abrahamsen A, Ekeberg Ø, Hannisdal E, Kaasa S. Psychological distress after cancer cure: a survey of 459 Hodgkin's disease survivors. Br J Cancer. 1997;76(6):791–796. https://doi.org/10.1038/bjc.1997.464
Thomas BC, Waller A, Malhi RL, et al. A longitudinal analysis of symptom clusters in cancer patients and their sociodemographic predictors. Journal of Pain and Symptom Management. 2014;47(3):566–578. https://doi.org/10.1016/j.jpainsymman.2013.04.007

**Table:**

Tumor location [included in model]
*Positive association with anxiety*
Enns A, Waller A, Groff SL, Bultz BD, Fung T, Carlson LE. Risk factors for continuous distress over a 12‐month period in newly diagnosed cancer outpatients. Journal of Psychosocial Oncology. 2013;31(5):489–506. https://doi.org/10.1080/07347332.2013.822052
The ACTION Study Group. Health‐related quality of life and psychological distress among cancer survivors in Southeast Asia: results from a longitudinal study in eight low‐ and middle‐income countries. *BMC Med*. 2017;15(1):10. https://doi.org/10.1186/s12916‐016‐0768‐2
